# Fellgett Revisited: On the Nature of Noise in Two-Dimensional Mass Spectrometry

**DOI:** 10.1021/jasms.4c00294

**Published:** 2024-10-25

**Authors:** Callan Littlejohn, Meng Li, Pui Yiu Lam, Mark P. Barrow, Peter B. O’Connor

**Affiliations:** † Department of Chemistry, University of Warwick, Gibbet Hill Road, Coventry CV4 7AL, United Kingdom; ‡ AS CDT, Senate House, University of Warwick, Coventry CV4 7AL, United Kingdom; § AMS-RTP, Millburn House, University of Warwick, Coventry CV4 7AL, United Kingdom

## Abstract

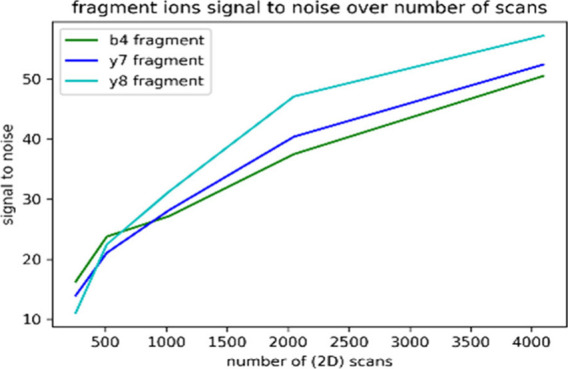

Two-dimensional mass spectrometry (2DMS) is a truly data-independent acquisition technique used in the analysis of complex mixtures; however, the nature of the noise within these spectra is not well understood. In this work, 2DMS is tested for conformity with the Fellgett principle: (signal/noise) ∝ √ (no. of data points). Since 2DMS functions through the modulation of ions through a fragmentation region across many scans, the individual scans are considered data points in this experiment. Random noise was shown to be prevalent as the main source of noise in this experiment with minor systematic noise. This means that the minimum size for a 2DMS spectrum that displays a target fragment ion can be determined using a fast-2D equation detailed herein. The effects of existing denoising algorithms were also found to change the relationship between the signal-to-noise ratio and the scan numbers to be of a quasi-linear nature rather than the square root trend observed before denoising.

## Introduction

**Figure 1:**
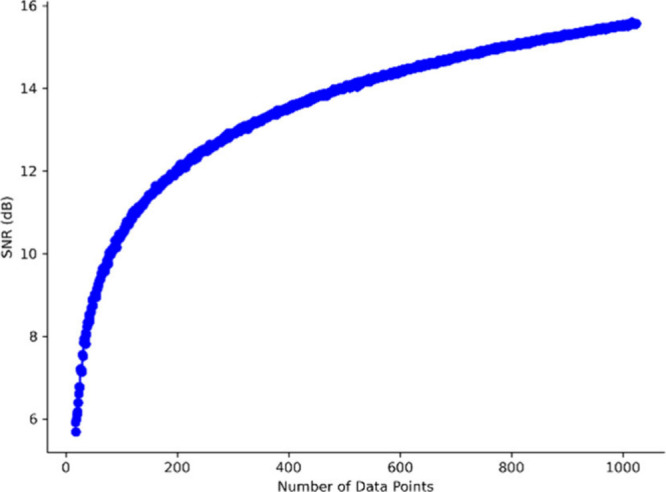
Modeled SNR of a signal with increasing number of data points showing an increase in SNR proportional to .

Noise is prevalent in all measurement-based systems. When this noise is nonreproducible and caused by spontaneous events, this is referred to as random noise. If a signal is stable and noise is random and if multiple measurements are taken and summed, the signal will increase linearly with the number of measurements while the noise will increase as the square root of the number of measurements, so that the signal-to-noise ratio will increase with the square root of the number of measurements. Furthermore, when the Fourier transform is used, this signal-averaging effect works for all signals in the spectrum simultaneously; this is because each sampling point is an independent sampling point for all possible signals. This principle is known as the Fellgett principle, named after its discoverer.^1^ In Fellgett’s work, the signal-to-noise ratio was always increasing with the square root of the number of sampling points for all signals in the spectrum simultaneously; a modeled example is included in Figure 1.

The Fellgett effect is well known and applied in many areas, including Fourier transform mass spectrometry (FTMS). In FTMS, sinusoidal signals generated by the movement of ions are correlated back to the mass to charge ratio (*m*/*z*) of the ions through the Fourier transform of the sinusoidal signals into frequency space followed by using calibration equations to convert frequency to *m*/*z*.^2−10^ In Fourier transform ion cyclotron resonance (FT-ICR), these sinusoidal signals are generated through the circular motion of ions known as cyclotron motion which occur when an ion is in motion within a strong magnetic field.^11−14^ The radius of this circular motion is generally increased through the use of RF electric potential frequency sweeps which excite ions to higher radial positions within an FT-ICR cell, which allows for the generation of coherence in ion packets and allows for phase-aligned signals to be detected corresponding to the ions’ cyclotron frequencies. FT-ICR has been used in the analysis of many complex mixtures and allows for routine high-resolution (∼3 million) studies to be conducted^15−22^ with ultrahigh resolution (15 M RP) possible under narrowband detection^23−25^ and further advances in techniques such as OCULAR leading to the possibility of constant high resolution across an entire spectrum.^26^ In FT-ICR experiments, the directly detected time domain signal generated by the sample is referred to as a transient, and these signals experience random thermal, Johnson–Nyquist noise^27,28^ primarily from the input resistor in the preamplifier^29−32^ and from ion current variations between scans in 2DMS.^33^ The length of a transient is determined by the number of data points divided by the sampling rate, where the sampling rate determines the lowest *m*/*z* possible to measure.^34^ It is therefore possible to increase the signal-to-noise of a peak by simply increasing the data set size, for example, by taking a longer transient while maintaining the same sampling frequency provided the signal is stable.^35^ Thus, increasing the number of data points in a transient increases signal averaging, which results in greater certainty of assignment for low-intensity peaks that may be lost to the noise at a low signal-to-noise ratio (i.e., with shorter transients). The detection of low-abundance signals is particularly important in fragmentation spectra, where some fragment ions may have a lower probability of being generated, which may lead to these being lost to the noise floor due to a low signal-to-noise ratio. In Fourier transform-based applications, having more data points allows for the additional benefit of increasing the resolution of the spectrum. As a result, lengthy, stable transients are usually preferred for analysis as they increase both the resolution and the signal/noise simultaneously.^36^ Although longer transients provide a benefit in terms of confidence in assignment, they also obviously require more time to acquire.

Two-dimensional mass spectrometry (2DMS) was first developed by Pfändler, Bodenhausen, Rapin, Houriet, and Gäumann in the late 1980s from pulse sequences adapted from 2D nuclear magnetic resonance (NMR.)^37^ The 2DMS experiment demonstrated correlation of fragment ions with their respective precursors without the need for isolation using a pulse–delay–pulse sequence which modulates ions at their cyclotron frequency through a fragmentation zone over a series of scans. The fragment ion intensity modulation is directly correlated to the precursor ion modulation through a spatially-dependent fragmentation zone, meaning that the intensity values in the final spectrum are correlated to the fragmentation efficiency of the precursor with the specific spatially-dependent fragmentation and the precursors and fragment ion signal intensities modulate at the cyclotron frequency of the precursor; therefore, all fragments of a given precursor will be found at the precursor ions frequency of fragmentation, which is able to be calibrated to its *m*/*z*. While mostly developed for use with FT-ICR MS, the technique has been applied on the linear ion trap^38^ and was recently demonstrated to be potentially possible in every mass analyzer.^39^

Two-dimensional mass spectrometry has shown significant advantages in the analysis of complex mixtures including a range of biomixtures,^40−44^ intact proteins,^16,45^ and polymers,^46^ showing precursors separated by millidaltons.^47^ The use of this truly data-independent acquisition technique allows for all of the fragment ion information to be obtained for a sample from a single spectrum, which could be a major benefit in clinical areas where a single spectrum could give the same information as performing multiple different tests. However, as with 1D studies using FTMS, the transient length of the acquisition means that these experiments can require 1 s or more to acquire and that the relatively slow acquisition rate compared to time-of-flight instruments is compounded in 2DMS as the experiment requires many scans to be taken so that experimental time scales on the order of tens of minutes are common. This time scale problem can be somewhat countered using larger magnets and new detection methods such as 2Ω.^48^

However, as a large number of scans are being taken and the FT is applied in both the precursor and the fragment dimensions, the signal-averaging advantage occurs in both dimensions. This study aims to determine whether the majority noise component of 2DMS in the vertical dimension is random or systematic and therefore explore the nature of noise and the Fellgett signal-averaging advantage in two-dimensional mass spectra.

**Figure 2:**
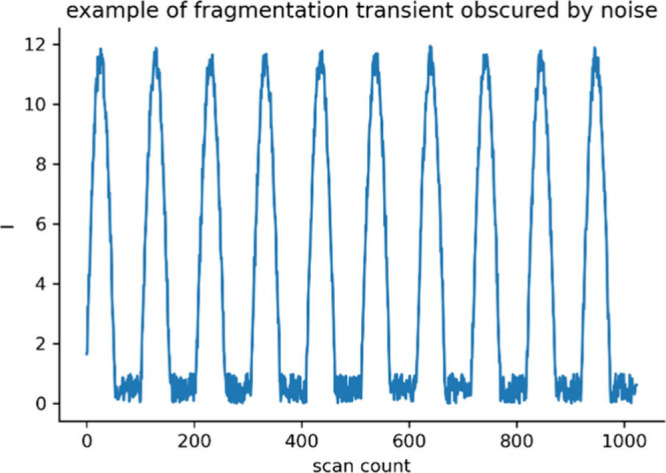
Example of fragmentation transients “cut off” by the noise floor.

In order to properly model noise, it is important to understand how modifications to a perfect transient effect the noise present within the transformed product. The Plancherel–Parseval theorem suggests that the energy before a FT should be equal to the energy after an FT. While Parseval’s theorem predates the FT,^49^ it was quickly applied to this analysis and was in common use by the 1930s.^50,51^ It may be more proper to refer to this adaptation as Plancherel’s theorem or the Plancherel–Parseval theorem after the more general work by Plancherel.^52^ This equivalence allows for the modeling of the signal-to-noise ratio to be significantly easier. For example, if the transient is split into a signal component and a noise component then the signal-to-noise ratio can be presented as
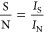
2where *I*_S_ and *I*_N_ are the intensity of the signal defined as the sum of the intensity of the signal, which means
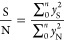
3where *y*_S_ is the *y* value of the signal at a given point and *y*_N_ is the *y* value of the noise at a given point. The use of Parseval’s theorem can help to simplify the modeling of noise. If a signal is generated with the equation
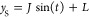
4where *J* is the amplitude of the wave and *L* is the offset from 0 then the power of the signal can be defined as
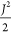
5This is the case when the entire wave is observed; however, in 2DMS, it is often the case that ion signals may drop below the noise floor. This is common because generally the excitation in 2DMS is tuned on one precursor peak, which means that it is not always possible to avoid clipping in all precursors. In this case, any contributions below the noise floor will not be counted toward the intensity of the signal as it is unobserved. This phenomenon can be described as asymmetric clipping, in that the signal is clipped on one or both sides independently of one another, an example of which is shown in Figure 2.

Signal clipping consistently degrades the signal quality and prominence. If the noise floor is defined at 0 and the offset *L* is the offset from the noise floor then finding the power of the resultant signal becomes a simple roots problem
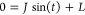
6
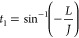
7
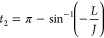
8
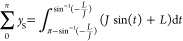
9which can be described in terms of scan count as
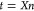
10
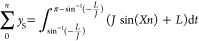
11which will estimate the total signal intensity in the precursor dimension.

When clipping occurs, however, the signal is not found in its pure form but instead tends to be split across various harmonics, which means that the power of the signal over the power of the noise does not necessarily equal the SNR of the observed peak. This effect can be measured using total harmonic distortion (THD) following the general trend
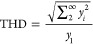
12where *y*_*i*_ is the amplitude of the *i*th harmonic and *y*_1_ is the amplitude of the fundamental frequency.

**Figure 3:**
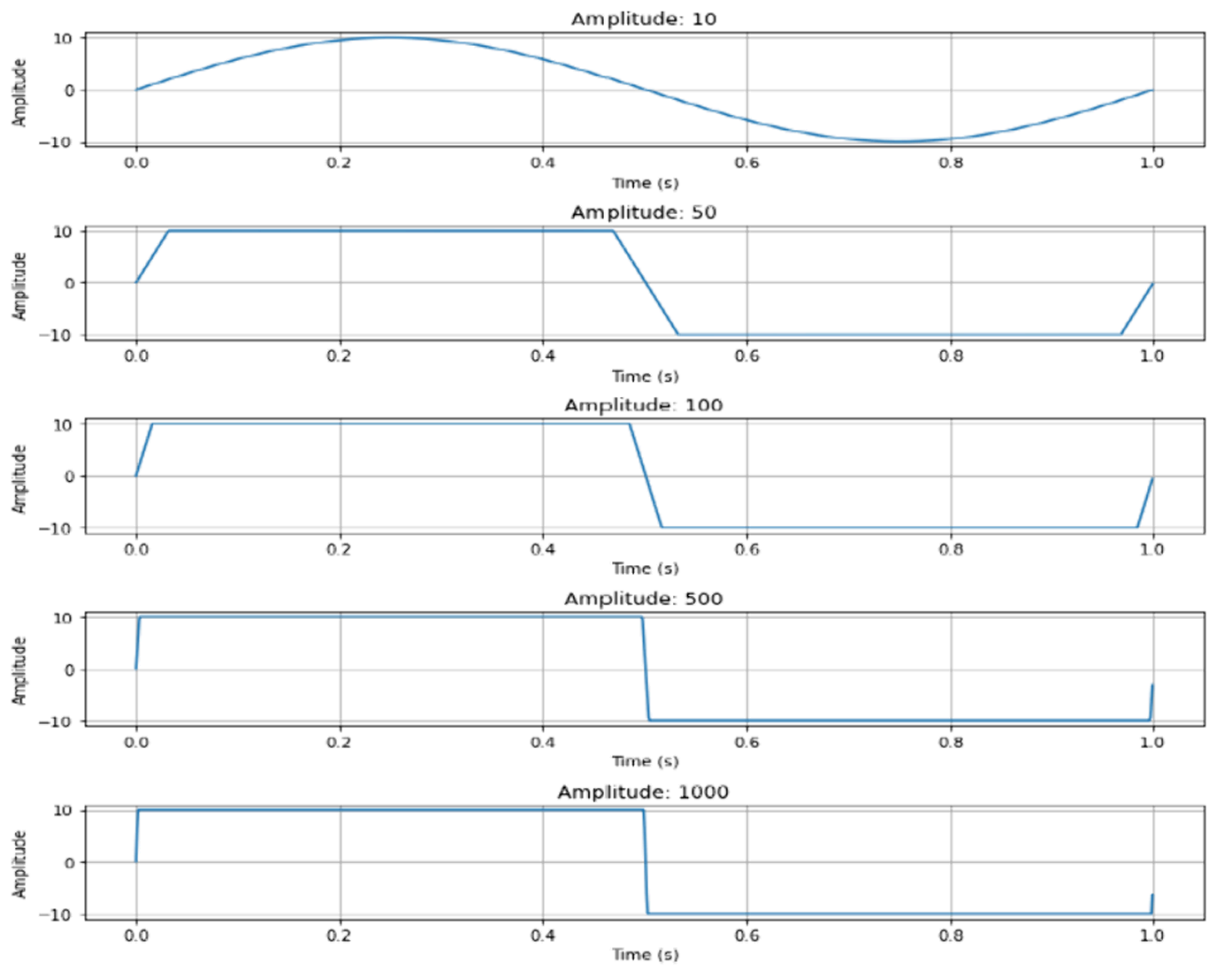
Symmetrically clipped waves tending toward square waves at high amplitude.

In simple cases, the THD can be calculated using the Cauchy method of resides; for example, a square wave has a value of 0.483, triangular waves a value of 0.121, and sawtooth a value of 0.803.^53^ This method becomes significantly more complex with less defined waveforms and in the case of the asymmetrically clipped wave becomes very difficult. For this reason, it is better to define the wave by its nearest likeness. It was found that symmetrically clipped waves can be considered to be analogous to the transition between sine waves and square waves. The higher the ratio of the amplitude to the clipping region is, the more the wave will resemble a square wave,^54^ as shown in Figure 3. This means that the clipped waveform THD should be between 0 and 0.483 for a symmetrically clipped waveform. Symmetrically clipped peaks do not exhibit even numbered harmonics, whereas asymmetrically clipped peaks do.

This allows for the creation of a final equation of the SNR in the precursor dimension of 2DMS defined as
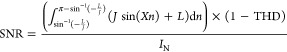
13By measuring the height of the ratio of the harmonics, it may be possible to back calculate the original unclipped waveform and increase the SNR of the fundamental peak.

## Methods

Agilent ESI tune mix, a mixture of oligomers of hexakis(fluoroxy)phosphazene, was purchased from Sigma-Aldrich (Merck Life Science Ltd., Dorset, UK) and used as an external calibrant. Bovine serum albumin (BSA) (A2153), formic acid (FA) (F0507), ammonium bicarbonate (ABC) (09830), dithiothreitol (DTT) (D-9779), iodoacetamide (IAA) (11149), and acetonitrile (ACN) (34851) were obtained from Sigma-Aldrich (Merck Life Science Ltd., Dorset UK). Trypsin (V5111) was obtained from Promega (Promega, WI, USA). Water was purified by a Millipore Direct-Q purification system (Merck Millipore, MA, USA).

A 6 μL amount of DTT (8 mg/mL) suspended in 100 mM ABC was added to 0.1 mg of BSA in 0.2 mL of 100 mM ABC and shaken at 1400 rpm and 60 °C using the Eppendorf Thermomixer C for 30 min. A 6 μL amount of IAA (19 mg/mL in 100 mM ABC) was then added to the resultant mixture and left in a dark cupboard for 60 min, after which 4 μL of trypsin (1 mg/mL in 100 mM ABC) was added and shaken at 37 °C for 16 h using the thermomixer. The digested protein was then collected at 1 mg/mL using a solaU C18 spin down column from Thermo-Fisher Scientific and diluted to 0.1 mg/mL in 49.5/49.5/1 ACN/H_2_O/FA for use in this experiment.

The solution was ionized using a home-built nanoelectrospray (n-ESI) source using emitters made from thin-walled glass capillaries (TW120F) from World Precision Instruments (World Precision Instruments Ltd. Hertfordhire, UK) pulled to a fine point using a Sutter P-97 flaming brown tip puller (Sutter Instruments CA, USA); the resultant tips were held with a potential of 800 V to the instrument entrance at a distance of approximately 2 mm. A Bruker 12 T solariX FT-ICR system (Bruker Daltonik GmbH, Bremen, Germany) was used throughout this study. Infrared multiphoton dissociation (IRMPD) fragmentation was achieved using a continuous wave, 25 W, CO_2_ laser (Synrad Inc., WA, USA) for 0.35 s at a laser power of 50%.

2DMS spectra of 256, 512, 1024, 2048, and 4096 scan lines were acquired with a scan acquisition of 1 M data points per transient and an acquisition rate of 1 Hz, resulting in a low-mass cutoff at *m*/*z* 147. The delay time was incrementally increased in 1.1 × 10^–6^ s time steps. The resultant transients were zero-filled once, apodized using a Kaiser apodization function,^55^ and processed using the Spike-Py processing software.^56^ Lines were then extracted using in-house software^40,47,57,58^ and exported to the Bruker Data Analysis program (Bruker Daltonik GmbH, Bremen, Germany) for signal-to-noise calculations, which appears to define the noise as 5× the standard deviation of points within a region of the spectrum after it has been smoothed with a Savitsky–Golay filter. All experiments were run on the same day with the same parameters and samples to ensure comparability.

**Figure 4:**
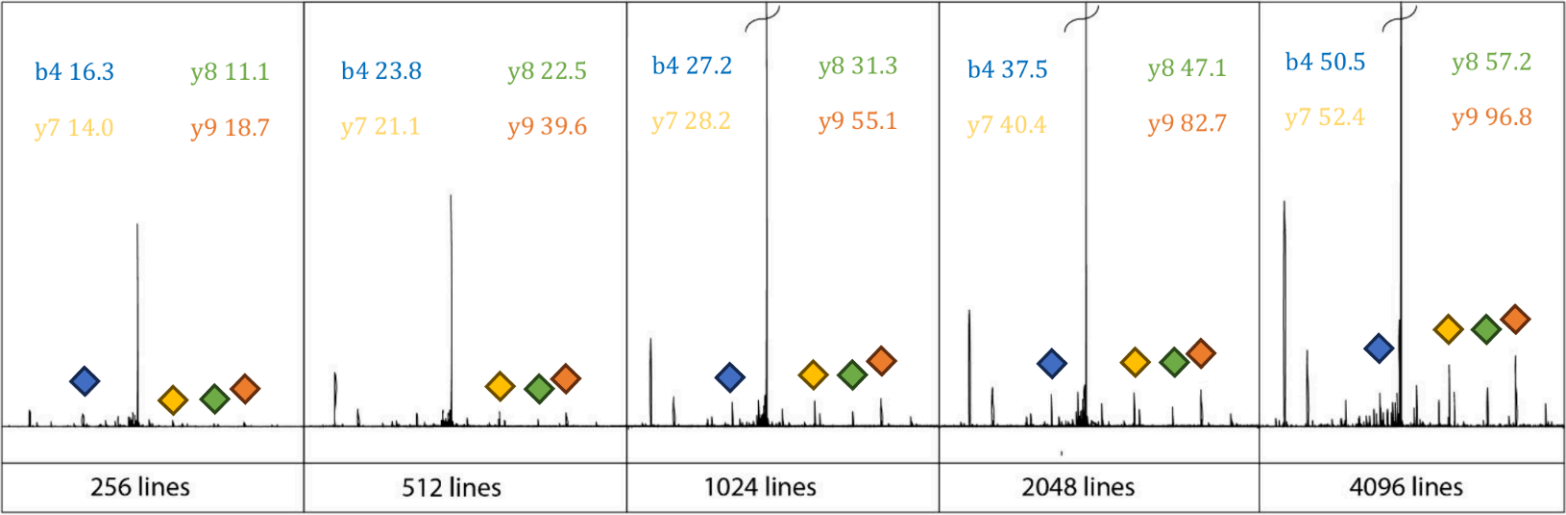
Lines extracted from *m*/*z* 653 of a 2DMS of BSA digest at different scan counts showing the increasing trend of the SNR with scan count.

After extracting the fragment line at precursor *m*/*z* 653.4 corresponding to the 2+ charge state of a BSA peptide (HLVDEPQNLIK), fragments of several peptides within the BSA digest were studied, as shown in Figure 4. The fragments of the *m*/*z* 653.4 precursor mass were chosen as the model example as they exhibited high intensity within the spectrum and therefore were more likely to be present at the lower scan numbers. Initial interrogation of the data showed a trend of increasing signal-to-noise ratio (SNR) with scan number, which is to be expected as increasing scan count is equivalent to increasing the length of the transient in the precursor dimension. Increasing the length of transient in data point space increases the signal-to-noise.^36^

## Results and Discussion

**Figure 5:**
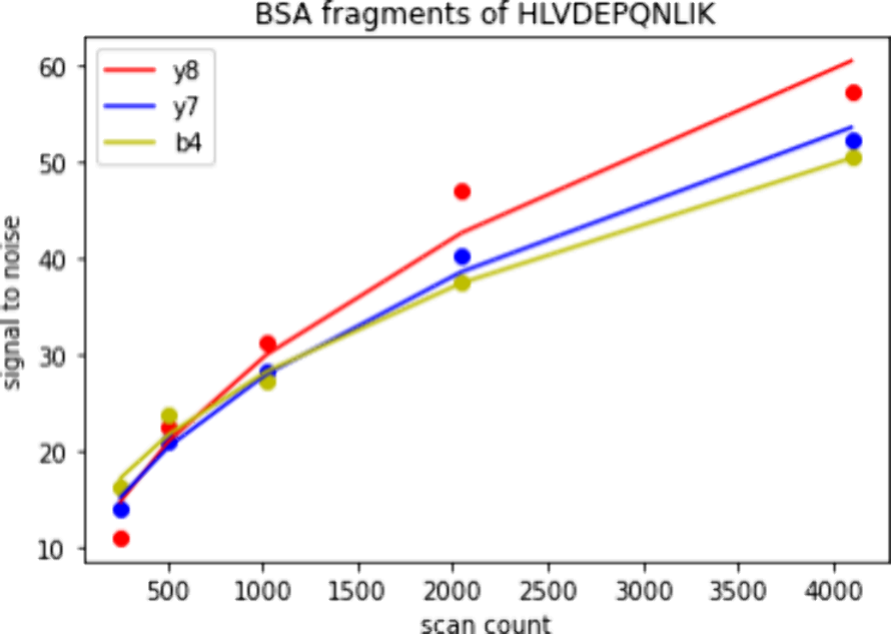
Increasing trend of the SNR with scan count shown in the fragments from the extracted line.

The Bruker Data Analysis program was used to determine the SNR. It was found that the trend of the SNR in relation to scan number was not linear but instead followed a function described in the equation
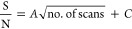
14where *A* and *C* are fitting constants. This trend is important as it constitutes the major component of the signal-to-noise ratio. The square root function shape suggests that the major component of the noise in the vertical dimension of a 2DMS experiment is random uncorrelated noise with a smaller component of continuous noise causing the presence of the “+c” term. This is shown in Figure 4, which shows the precursor scan line corresponding to *m*/*z* 653.4. As is clear from the graph in Figure 5 that the relationship is certainly not linear and can be shown to follow the same trend as one would expect of the equation above. The parameters for each curve fit are shown in SI Table 1.

It is important to note that the equation is not the same for all fragments. This is unintuitive until the parameters of this experiment are taken into account. One critical aspect of adherence to the Fellgett principle is that the signal is present at every sampling point. In 2DMS, this is not necessarily always true if the fragmentation intensity at a given excited radius is not strong enough to overcome the energy requirement to produce a certain fragment. In such a case, the fragment will not appear in that particular scan, which leads to several scans where the bottom of the fragmentation transient is essentially “chopped off” by the noise floor. If the derivation in the introduction is combined with the Fellgett principle, it is obvious that these additional parameters are required to account for the clipping and the harmonic component
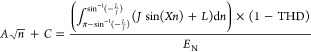
15Rearranging to
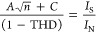
16which means that intensity per scan can be determined as shown in Table 1. More information would be required to access the values for *J* and *L* in this equation; however, comparing the intensity per scan to the ideal average intensity per scan can provide insights into the 2DMS experiment. With this in mind, the fitting parameters shown in SI Table 1 can now be understood more clearly. This relationship can also be used to tune a 2DMS as the less optimized the 2DMS is in terms of overlap with the fragmentation technique, the higher the contribution of *D* in this equation.

**Table 1:** Calculated THD and SNR per Scan

fragment	THD	(*E*_S_/*E*_N_)/scan
Y7	0.205	4.127
Y8	0.118	0.635
Y9	0.113	2.706
B4	0.247	9.160

One of the main limitations of two-dimensional mass spectrometry is that due to the high number of scans usually required for complex mixture analysis coupled with the high temporal cost of gaining an FT-ICR transient, the time to generate 2DMS spectra often ranges from 20 min to 4 h. Coupled with the processing and analysis requirements for these huge data sets (several GB to TB of data), 2DMS in its current form is inaccessible for many clinical or nonresearch-based settings where speed and throughput of samples are of importance. With the knowledge of the SNR trend with scan numbers, it is possible to determine the minimum required number of scans to observe a specific fragmentation phenomenon. For example, in Figure 5, it is possible to see the rising SNR of the fragments; however, all fragments studied were still present at 256 scan lines, which takes roughly 4 min to acquire. This speed would certainly allow for the fast acquisition of 2DMS with a reduced signal-to-noise ratio and resolution for analytical or data-based confirmatory measures. Additionally, as 2DMS is a truly a data-independent acquisition technique, all of the data contained within the 4096 scan lines in 2DMS are also present in the 256 scan lines but with different resolutions in the precursor dimension and with a significantly lower signal-to-noise ratio.

This trend allows the user to estimate the number of scans necessary to gain the information required from the experiment. For example, if the resolution is not a key factor in the precursor dimension (i.e., less complex mixtures) and the fragmentation efficiency of the analytes is relatively high (2DMS benefits from high fragmentation efficiency as this increases the magnitude of the sinusoidal relationship which is needed for correlation)^59^ then it would be possible to perform a shorter 2DMS and still maintain the wealth of information from within the spectrum. Due to the use of a fast Fourier transform (FFT), the scan numbers traditionally must be limited to powers of 2 in order to maintain efficiency with the Cooley–Tukey algorithm,^60^ which means that the user has to decide if a √2-fold signal increase is worth doubling the acquisition time.

### Calculating the Required Number of Scans in a 2DMS

In order to optimize the necessary scan count of any 2DMS, it is first important to ascertain what SNR should be expected. The most efficient way is to complete the experiment once with a large number of scans on a standard similar to or identical with the analyte. After this has been done, target peptides and fragments can be located within the 2DMS spectrum and their signal-to-noise ratio determined. Once the signal-to-noise ratio has been determined, the calculation becomes trivial as shown in eq 3
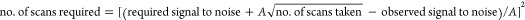
17This equation can be used to calculate the required scan lines in order to perform a useful 2DMS experiment. For example, when the 2DMS spectra discussed above were artificially cut by only processing a certain number of the acquired spectra, simulating a shorter acquisition, the B4 fragment was present in the resultant 2DMS in a 2DMS as small as 64 scan lines; the theoretical minimum using the above equation is 55 scans. If a 15 SNR is needed, a 2DMS spectrum of a similar standard that exhibits similar fragmentation efficiency showed a SNR of 50 at the target. The required scan numbers would appear to be around 368; however, due to the nature of the FFT, 512 scan lines should be sufficient in this case. Using a similar or comparable standard is possible because the intensities of the peaks within the 2DMS spectrum are linearly dependent on the average amplitude of modulation of the fragment intensity within the transients by definition. Thus, an ion with a higher modulation intensity across the 2DMS experiment will exhibit higher intensity in the spectrum, whereas ions which do not fragment will not appear in the spectrum. This is however uncommon. While the speed of acquisition is largely defined by instrument parameters such as transient length, optimization of the number of scan lines necessary could result in a decrease in acquisition time by reducing the number of scans taken, albeit with a concomitant decrease in the vertical, precursor-ion resolution and would be significant in a clinical setting with as many as 8 or 9 samples being run in the space of time it would take to run 1 in the longer method.

### Effects of Denoising Algorithms on Signal Averaging Effects

**Figure 6:**
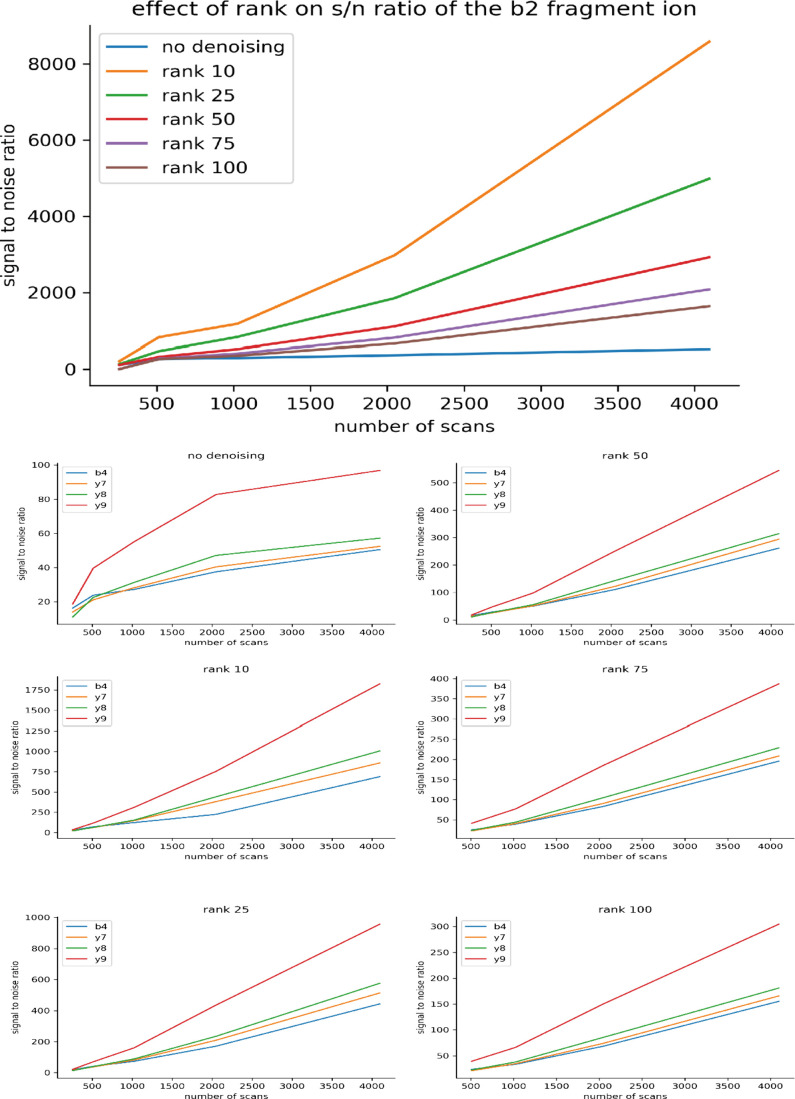
Graphs showing the effect of denoising on the fragments from the extracted line.

In 2DMS processing, a common method of boosting the signal-to-noise ratio is through denoising algorithms such as uRQRD or SANE,^61^ so it is important to determine the effect that these algorithms have on the signal-to-noise gain of a 2DMS experiment. The same data set as before was reprocessed with the uRQRD algorithm applied between the two Fourier transforms, i.e., before the fragmentation transients were Fourier transformed. The results were then interrogated in an identical manner. The values of signal-to-noise ratio for fragment peaks within the *m*/*z* 653.4 precursor line of BSA digest 2DMS spectra ranging from 128 to 4096 scan lines were plotted with the signal-to-noise ratio in relation to the scan count, and the relationship was almost linear as shown in Figure 6. This linearity likely comes from the denoising algorithm. Close inspection of the uRQRD algorithm suggests that at some point the data are, essentially, multiplied by a component of itself. This is confirmed in the source code for uRQRD where the value output is the identity matrix of random numbers convolved with the data multiplied by the data set itself.^61^ This effect is very similar to simply processing the data, where the final intensity values are all squared. This produces a very similar looking spectrum with very similar signal-to-noise ratios; however, it is important to notice the difference between uRQRD which does denoise the data and squaring the data does not.

One important input into the uRQRD algorithm is rank, the effect of which can be seen in Figure 6. Rank is set experimentally to equal the amount of expected peaks and practically determines how many instances of the convolved data are averaged with each other. The effect of rank on this signal-to-noise ratio was also investigated, and some interesting trends were noted. First, as expected from the theory of uRQRD, the signal-to-noise ratio rises with decreasing rank. And, the relationship is linear across ranks. However, the smaller size 2DMS spectrum around 256 and 128 scan lines exhibits loss of some low-abundance fragment ions when denoised with a high rank (50–100). Due to their presence in the nondenoised spectrum with reasonable signal to noise (∼10–30), this effect is attributed to the denoising algorithm and is expected when denoising is applied before an FT as denoising may act to smooth any waves which might be present in the signal. If this wave is of low amplitude, as would be expected in fragments of low intensity, then the wave may be smoothed to below the random noise level. This effect will be true for all denoising algorithms working in this fashion, and as such, denoising should be handled with care in 2DMS processing.

The additional processing time compounded with the loss of low-abundance peaks suggest that any denoising algorithm should be used with care in detailed analysis due to the possibility that they could distort peak intensities and lead to potential loss of low-intensity peaks, which may add to the difficulty in analysis. The processing affects the analysis, and therefore, consideration must be given to the observation that denoised 2DMS studies appear to be biased toward the high-intensity peaks. Other potential losses of signal may include signal suppression and space charge, which may cause larger effects than that of denoising depending on the experimental conditions.

For this reason, it is strongly recommended that 2DMS data without denoising should be processed before deciding if denoising is in fact necessary for the analysis or presentation of the data.

## Conclusions

A major element of noise within two-dimensional mass spectrometry is thermal Johnson–Nyquist noise, which is completely random and uncorrelated from measurement to measurement. It has been shown in 2DMS that due to this random noise, the SNR increases with the square root of the number of data points in each transient as well as in the number of individual scan lines acquired in the vertical modulation dimension provided the signal is stable. As a result of this trend, it is possible to optimize the size of acquisition for any given 2DMS spectrum based on the required SNR. This should allow for faster acquisition of 2DMS data and allow for a wider application of the technique to other fields such as clinical use.

Clinical applications of 2DMS are potentially powerful as this technique is completely data independent, allowing for the possibility of performing a multitude of tests in one experiment and, more importantly for the patient, using one sample.

The effect of the scan number on the signal-to-noise ratio was also tested using a denoising algorithm. It was found that the correlation was more linear (potentially supralinear) compared to those without denoising; however, at low scan numbers, some low-intensity fragment peaks disappeared at lower scan numbers than expected. Additionally, it was determined that in terms of speed, current denoising algorithms are problematic. For this reason, it was suggested that a better solution would be acquiring larger data sets, which would help both the SNR as well as the resolution, and denoising can always be applied retrospectively if needed.

## Supporting Information Available

Fitting constants for each fragment ion studied, deeper discussion on noise calculations, and modeling of asymmetric clipping (PDF)
