# Supplementary material for: Fellgett Revisited: On the Nature of Noise in Two-Dimensional Mass Spectrometry
Source: J Am Soc Mass Spectrom. 2024 Oct 25;35(12):2984–92. doi: 10.1021/jasms.4c00294 (PMC11622379; doi:10.1021/jasms.4c00294)
Supplement: Supplementary file 1 — js4c00294_si_001.pdf [file js4c00294_si_001.pdf]

Supporting Information

Fellgett revisited: On the nature of noise in Two-Dimensional Mass Spectrometry.

Callan Littlejohn[1,2], Meng Li[3], Pui Yiu Lam[1], Mark P. Barrow[1], Peter B. O'Connor[1,3]*

1 Department of Chemistry, University of Warwick, Gibbet Hill Road, Coventry, CV4 7AL, UK

2 AS CDT, Senate House, University of Warwick, Coventry, CV4 7AL, UK

[3] AMS-RTP, Millburn House, University of Warwick, Coventry, CV4 7AL, UK

*corresponding author

Fragment	A fitted	C fitted	R2
y7	0.799	2.482	0.993
y8	0.951	-0.391	0.965
y9	1.607	0.794	0.948
b4	0.691	6.207	0.991

Table SI1: Fitting constants for each fragment ion studied

Noise calculations

The Bruker Dataanalysis help files suggest that the noise of a spectrum is calculated using the Noise method within Dataanalysis. The method calculates noise by the following method taken from the help files:

The third derivative of the spectrum is calculated using a Savizky-Golay filter, using a smoothing width of 1 this simplifies to:

$$y_i' = \frac{1}{2}y_{i+1} - \frac{1}{2}y_{i-1}$$

The standard deviation, σ, of all values of the third derivative within a set region is determined

$$\sigma = \sqrt{\sum_{i=1}^{N} \frac{y_i'''^2}{N}}$$

The signal to noise of a peak is then calculated as:

$$SNR = \frac{S}{5\sigma} \quad S = peak\ height\ above\ baseline$$

Bruker estimates that 99% of noise values are within 5σ.

Asymmetric clipping modelling

A Model was generated to attempt to understand the effect of asymmetric clipping on the signal intensity. Figure SI1 shows that the intensity of the signal appears to follow a pseudo sinusoidal relationship with L, and this is because as L increases there is a progressive loss in the integral (area under the curve) of the time domain signal which translates to a loss in overall signal intensity. A the signal is periodic and sinusoidal the relationship also follows the same pattern.

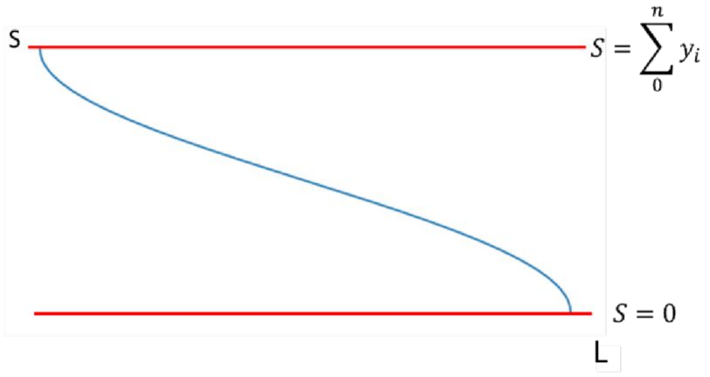

Figure SI1: Modelled signal intensity showing a decrease in signal intensity with increasing clipping
